# A Fast CT Reconstruction Scheme for a General Multi-Core PC

**DOI:** 10.1155/2007/29160

**Published:** 2007-07-09

**Authors:** Kai Zeng, Erwei Bai, Ge Wang

**Affiliations:** ^1^Biomedical Engineering Department, University of Iowa, Iowa City, IA 52244, USA; ^2^Electrical and Computer Engineering Department, University of Iowa, Iowa City, IA 52244, USA; ^3^Biomedical Imaging Division, VT-WFU School of Biomedical Engineering and Sciences, Virginia Polytechnic Institute and State University, Blacksburg, VA 24061, USA

## Abstract

Expensive computational cost is a severe limitation in CT reconstruction for clinical applications that need real-time feedback. A primary example is bolus-chasing computed tomography (CT) angiography (BCA) that we have been developing for the past several years. To accelerate the reconstruction process using the filtered backprojection (FBP) method, specialized hardware or graphics cards can be used. However, specialized hardware is expensive and not flexible. The graphics processing unit (GPU) in a current graphic card can only reconstruct images in a reduced precision and is not easy to program. In this paper, an acceleration scheme is proposed based on a multi-core PC. In the proposed scheme, several techniques are integrated, including utilization of geometric symmetry, optimization of data structures, single-instruction multiple-data (SIMD) processing, multithreaded computation, and an Intel C++ compilier. Our scheme maintains the original precision and involves no data exchange between the GPU and CPU. The merits of our scheme are demonstrated in numerical experiments against the traditional implementation. Our scheme achieves a speedup of about 40, which can be further improved by several folds using the latest quad-core processors.

## 1. INTRODUCTION

CT imaging has seen tremendous development over the past decades. Now, it is widely used in the medical imaging field. However, due to the high computational cost required for reconstruction, its real-time imaging applications [1] remain challenging. Bolus-chasing computed tomography (CT) angiography is a primary example which demands real-time CT feedback.

To address this problem, various techniques are used for fast image reconstruction. A number of commercial hardware-based solutions are available. For example, XTrillion (by TeraRecon, Inc.) uses an application-specific PCI card, while Mercury Computer Systems relies on blade-based Linux clusters. However, the specialized hardware is expensive and unsuitable for general purpose applications. Alternatively, efforts are made using graphic cards [2, 3], since the main operation for commercial CT reconstruction is backprojection, similar to texture mapping in computer graphics [4]. Although graphics cards are highly optimized, they do not support floating-point calculations. Hence, they are not ideal for medical imaging applications. Despite that the latest graphics cards can implement virtual floating-point calculations [3, 5], they do not support full 32 bits floating calculations. Another bottle-neck is that the graphic cards require data exchange between CPU and GPU.

In this paper, a multi-core PC-based acceleration scheme is proposed for filtered-backprojection-(FBP-) based image reconstruction. This scheme reduces computational cost and maintains image quality. Our scheme integrates the following techniques for fast image reconstruction. First, geometric symmetry is taken into account to eliminate redundant operations. That is, only one computation is performed for multiple symmetric positions. Second, efficient data structures are used to minimize the data access time. Third, the single-instruction multiple-data (SIMD) technique is employed for data-level parallel processing. Fourth, the multithreading programing is done to take advantage of multi-core processors, realizing the true parallel computation capability. Finally, an Intel C++ complier is used to optimize the code for Intel processors.

This paper is organized as follows. In Section 2, the CT reconstruction algorithm is overviewed, and then each of our acceleration techniques is described. In Section 3, numerical experiments on various datasets and different PCs are presented to evaluate the speedups with our scheme and the conventional implementation. In Section 4, relevant issues and research directions are discussed.

## 2. MATERIAL AND METHODS

### 2.1. CT reconstruction algorithm

The most popular multislice CT reconstruction methods remain data rebinning-based fan-beam reconstruction filtered backprojection (FBP) algorithms. Therefore, our work is focused on the typical fan-beam FBP algorithm. Note that the application of our scheme is not limited to the fan-beam case, because it can also be applied to accelerate the latest approximate cone-beam algorithms [6–8], which can be treated as generalized fan-beam reconstruction algorithms.

**Figure 1:**
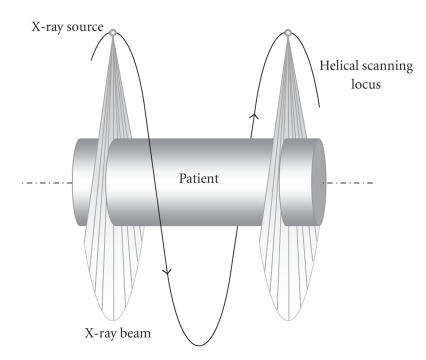
Scanning geometry with the patient static and source moving in a helical trajectory.

**Figure 2:**
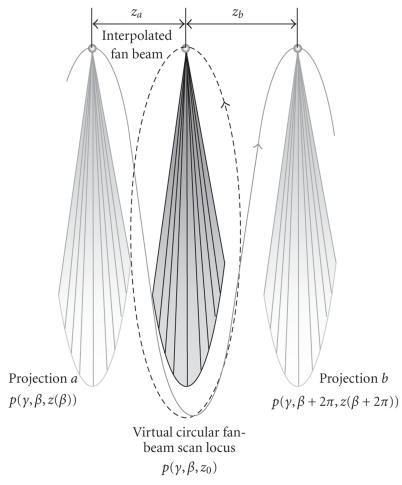
Helical data interpolation scheme. Helical scanning projection data are rebinned into a series of circular scan datasets via linear interpolation.

**Figure 3:**
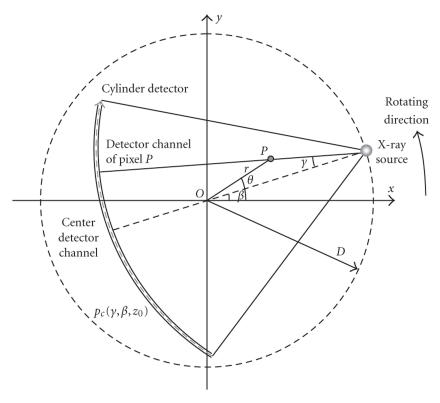
Fan-beam geometry on the *z* = *z*_0_ plane.

In a typical CT setting, the data acquisition system (an X-ray source and a detector assembly) is rotated rapidly in the gantry while the patient on a table is translated into the gantry opening. This process is illustrated in Figure 1. Because the multi-row detector arrays span a very small cone angle, acquired helical scan data are usually rebinned into a series of virtual circular scan data for reconstruction of a stack of images [9]. Here we assume a method from [10], in which the virtual fan-beam projection data are calculated according to the following formula:

(1) $$p_{c}\;\left(\gamma ,\beta ,z_{0}\right)=p_{h}\left(\gamma ,\beta^{\prime },z\left(\beta^{\prime }\right)\right)\frac{z_{b}}{z_{a}\;+z_{b}\;}+p_{h}\left(\gamma ,\beta^{\prime }+2\pi ,z\left(\beta^{\prime }+2\pi \right)\right)\frac{z_{a}}{z_{a}\;+z_{b}\;},$$

where *β*′ = *β* + *k* × 2 *π*, *k* ∈ *N*, so that *z*_*a*_ ≤ *z*_0_≤ *z*_*b*_, *p*_*c*_ denotes virtual circular scan data, *p*_*h*_ denotes acquired helical projection, *z*_*a*_ and *z*_*b*_ are the distances from projections *a* and *b* to the virtual circular plane, respectively, in Figure 2, *β* and *γ* are the projection angles shown in Figure 3.

**Figure 4:**
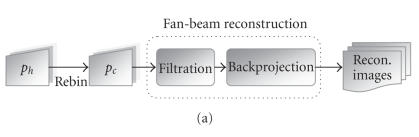

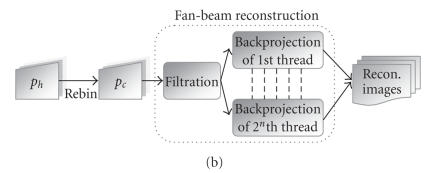
Flowchart of the helical CT reconstruction algorithm: (a) process with one thread, and (b) with multithreads.

After transforming helical projection data *p*_*h*_ to circular fan-beam projection data *p*_*c*_, the conventional fan-beam reconstruction algorithm [11] can be used. As the rebinning cost is insignificant, our optimization targets the reconstruction process:

(2a) $$f\left(x,y,z_{0}\right)=\int_{0}^{2\pi }\frac{1}{L^{2}}p_{c,f}\left(\gamma_{0},\beta ,z_{0}\right)d\beta ,$$

(2b) $$p_{c,f}\left(\gamma ,\beta ,z_{0}\right)=p_{c}\left(\gamma ,\beta ,z_{0}\right)D\text{cos }\gamma *\frac{\gamma^{2}}{2{\text{sin}}^{2}\gamma }h(\gamma ),$$

(2c) $$L=\sqrt{D^{2}+r^{2}-2Dr\text{cos}\left(\beta -\theta \right)},$$

(2d) $$\gamma_{\text{0}}\text{ =arcsin}\frac{r\text{sin}\left(\theta -\beta \right)\;}{L},$$

where *f* is an object function to be reconstructed, *p*_*c*, *f*_ are the filtered projection data, *D* is the distance from the source to the center of rotation, and *h*(*γ*) is the ramp filter [11]. While the inner convolution is the filtration process, the outer integration is the most time-consuming backprojection process, as shown in Figure 4(a).

### 2.2. Acceleration techniques

Since the backprojection is the bottleneck, let us analyze the backprojection process as shown in Algorithm 1. Clearly, a large part of the computational cost is due to the inner loop that calculates *γ*_0_, 1/*L* ^2^, interpolation coefficients, and accumulates the incremental contributions to the final reconstruction. In the following, we show how the backprojection can be speeded up using various techniques.

#### 2.2.1. Utilization of geometric symmetry

**Figure 5:**
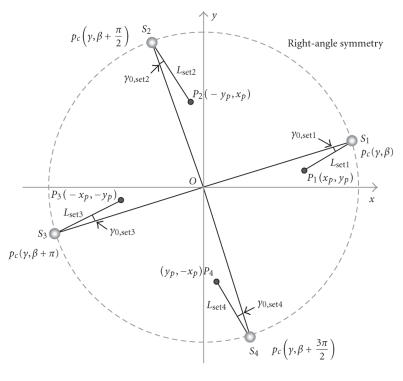
Right-angle symmetry. Four pairs of source and pixel positions share the same *L* and *y*_0_.

For our circular fan-beam reconstruction, two types of symmetries are available, which are referred to as the right-angle symmetry and complement symmetry. The right-angle symmetry, or 90-degree symmetry, is shown in Figure 5. That is, a new pair of source and pixel positions is obtained by applying a 90-degree rotation to a current pair of source and pixel positions. The resultant 4 pairs of source and pixel positions share the same 1/*L* ^2^ and *γ*_0_, which can be calculated from (3) and (4), respectively. As the interpolation coefficients required by the backprojection are determined by *γ*_0_, they are the same as well. Therefore, for the four sets under consideration, the calculations of these parameters need to be done only once:

(3) $$\begin{array}{c} L_{\text{set}1}\;=\sqrt{D^{2}+r^{2}-2Dr\cos\;\left(\beta -\theta \right)}\\ =\sqrt{D^{2}+\left(x_{p}^{2}+y_{p}^{2}\right)-2Dr\cos\left(\beta -\tan^{-1}\left(x_{p},y_{p}\right)\right)}\\ =\sqrt{D^{2}+\left(y_{p}^{2}+x_{p}^{2}\right)-2Dr\cos\left(\beta +\frac{\pi }{2}-\tan^{-1}\left(y_{p},-x_{p}\right)\right)}\\ =\;L_{\text{set2}}\;\\ =\sqrt{D^{2}+\left(x_{p}^{2}+y_{p}^{2}\right)-2Dr\cos\left(\beta +\pi -\tan^{-1}\left(-x_{p},-y_{p}\right)\right)}\\ =\;L_{\text{set3}}\\ =\sqrt{D^{2}+\left(y_{p}^{2}+x_{p}^{2}\right)-2Dr\cos\left(\beta +\frac{3\pi }{2}-\tan^{-1}\left(-y_{p},x_{p}\right)\right)}\\ =\;L_{\text{set4}}, \end{array}$$

(4) $$\begin{array}{c} \gamma_{0,\text{set}1}=\arcsin\frac{r\sin\left(\tan^{-1}\left(x_{p},y_{p}\right)-\beta \right)}{L}\\ =\arcsin\frac{r\sin\left(\tan^{-1}\left(-y_{p},x_{p}\right)-\beta -\pi /2\right)}{L}\\ =\gamma_{0,\text{set}2}\\ =\arcsin\frac{r\sin\left(\tan^{-1}\left(-x_{p},y_{p}\right)-\beta -\pi \right)}{L}\\ =\gamma_{0,\text{set3}}\\ =\arcsin\frac{r\sin\left(\tan^{-1}\left(-y_{p},x_{p}\right)-\beta -3\pi /2\right)}{L}\;\\ =\gamma_{0\text{set4}}, \end{array}$$

where (*x*_*p*_, *y*_*p*_) denotes the pixel in the first quadrant. The following two requirements, which are usually satisfied in practice, are necessary to use the right-angle symmetry. The first requirement is that the projection data must be available at the involved four angles. Namely, the number of projections in a full scan must be divisible by 4, which is reasonable for current medical CT scanners. For instance, a SOMATOM system generates 1160 projections per turn, while a Lightspeed scanner produces 984 projections per turn. The other requirement is that the reconstruction region must be symmetric about the *x*- and *y*-axes, such as a square or a circle in the clinical imaging situation.

**Figure 6:**
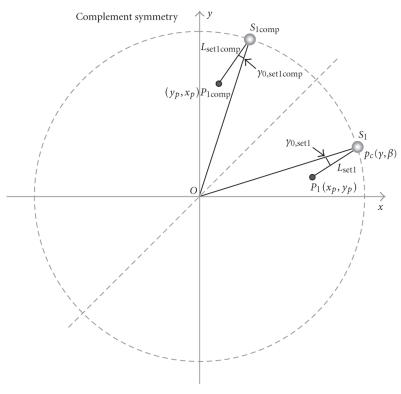
Complement symmetry. Two pairs of source and pixel positions share essentially the same *L* and *y*_0_.

The second type of symmetry is the complement symmetry, as shown in Figure 6. Here, a pair of source and pixel positions complements the other pair of source and pixel positions if they are symmetric with respect to a diagonal line (e.g., *y* = *x*). For these 2 pairs of source and pixel positions, *L* s are the same, while *γ*_0_ for one has the opposite sign of that for the other, as shown by (5) and (6), respectively,

(5) $$\begin{array}{c} L_{\text{set}1}\;=\sqrt{D^{2}+r^{2}-2Dr\cos\;\left(\beta -\theta \right)}\\ =\sqrt{D^{2}+\left(x_{p}^{2}+y_{p}^{2}\right)-2Dr\cos\left(\beta -\tan^{-1}\left(x_{p},y_{p}\right)\right)}\\ =\sqrt{D^{2}+\left(y_{p}^{2}+x_{p}^{2}\right)-2Dr\cos\left(\frac{\pi }{2}-\beta -\tan^{-1}\left(y_{p},x_{p}\right)\right)}\\ =\;L_{\text{set2c}}, \end{array}$$

(6) $$\begin{array}{c} \gamma_{0,\text{set}1}=\arcsin\frac{r\sin\left(\tan^{-1}\left(x_{p},y_{p}\right)-\beta \right)}{L}\\ =\arcsin\frac{r\sin\left(\tan^{-1}\left(y_{p},x_{p}\right)-\left(\pi /2-\beta \right)\right)}{L}\\ =-\gamma_{0,\text{set1c}}. \end{array}$$

Therefore, such a symmetry can also be used to reduce the computational cost. The requirements for use of the complement symmetry are the same as those for the right-angle symmetry.

Using these two types of symmetries, the backprojection can be significantly speeded up, since only one set of parameters needs to be calculated for the eight sets. The implementation of the backprojection is accordingly modified, as shown in Algorithm 2. Note that after the calculation of *γ*_0_ and *L* once for 8 pairs of source and detector positions, 8 filtered projection values are put to 8-pixel positions together in the inner-loop.

#### 2.2.2. Optimization of data structures

**Figure 7:**
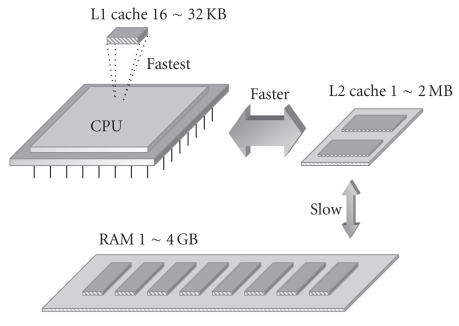
Mechanism for CPU to access data via multilevel caches.

To evaluate the computational complexity, the time for CPU to access data must be considered, especially for the CT reconstruction process because the backprojection requires frequent visits to a great amount of filtered projection and image data. The CPU data access mechanism with multi-level caches is illustrated in Figure 7. Specifically, a cache can be used to reduce the average time to access data in the main memory (RAM). The cache is a smaller, faster memory chip which stores copies of data from the most frequently used main memory locations. As long as a majority of memory accesses are to the cached memory locations, the average latency of memory accesses will be reduced to the cache latency, instead of the main memory latency. The L1 cache is the fastest and usually about 16 ∼ 32 KB. The L2 cache is faster than RAM and about 1 ∼ 2 MB. The slowest RAM is 1 ∼ 4 GB.

When the processor needs to read from or write to the main memory, it first checks if the data is in the cache. If it is in the cache, we say that a cache hit has occurred; otherwise a cache miss is counted. In the case of a cache hit, the processor immediately reads or writes the data. However, in the case of a cache miss, it takes much longer time to access the data. Due to the limited cache capacity, one way to execute the code efficiently is to increase the hit rate by optimizing the data structures.

**Figure 8:**
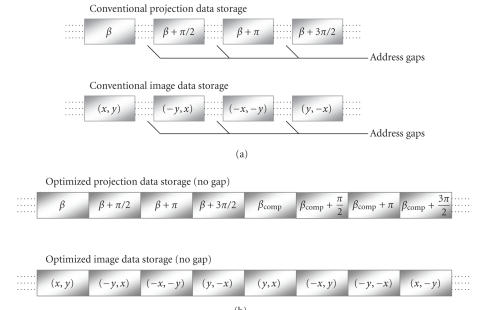
Conventional and our data storage structures: (a) conventional data storage scheme and (b) our optimized storage scheme.

Usually, projection data are sequentially stored in the order of *β*, while reconstructed images are stored rowwisely. Thus, for implementation of the right-angle and symmetry, the access to 8 pairs of projection and image data will very likely result in cache misses due to the address gaps, as shown in Figure 8. Such misses within the inner loop will cause a significant latency. To address this problem, in our optimized data structures all the data are arranged into blocks indexed to reflect symmetric relationships. Therefore, the cache miss rate can be greatly reduced in the inner loop.

#### 2.2.3. SIMD technique

**Figure 9:**
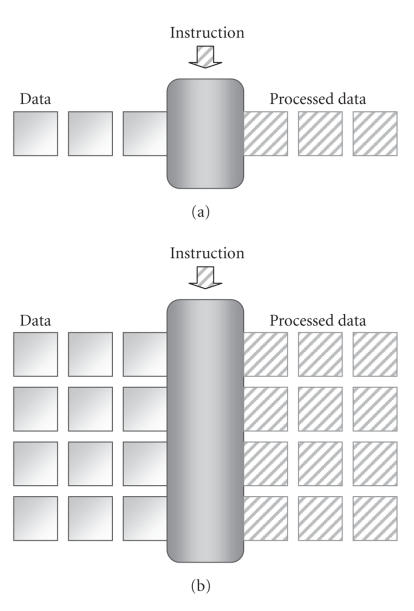
SISD and SIMD techniques: (a) SISD and (b) SIMD.

The SIMD technique enables the data-level parallelism like in a vector processor, as shown in Figure 9. With an SIMD processor, one instruction can process a block of data at a time instead of just one datum, which is much more efficient than the conventional single instruction single-data (SISD) technique. Small-scale (64 or 128 bits) SIMD operations are now popular supported by general PC CPUs, such as those from Intel and AMD [12, 13]. We use the Intel SSE (streaming SIMD extensions) instruction set to implement the SIMD technique in our backprojection process. Within the inner loop, we backproject 8 projection data onto 8 pixels, according to the same instructions such as interpolation, weighting, and accumulation. Therefore, we have a perfect situation to employ the SIMD technique. As the SSE only supports simultaneous processing of 4 floating data at a time (128-bits register), 8 data are processed in two groups.

#### 2.2.4. Multithreaded programing

In recent years, the great increment of the clock speed of PC processors seems difficult. Intel is bounded by 4 GHz, while AMD stays under 3 GHz. Their efforts have now shifted from improving the clock speed to increasing the number of cores within a processor. Dual-core quad-core processors become commercially available for a PC. However, a processor with more than one core cannot achieve a better performance unless parallel computation schemes are applied. Therefore, to take advantage of multi-core processors, multi-threaded programing must be done.

From the flowchart of our algorithm, the computation within the inner loop is independent. Thus, the backprojection can be implemented in parallel by assigning different loop ranges to various cores of the processor. After all the threads are finished, the final result can be assembled from the results of each thread. In our implementation (Figure 4(b) and Algorithm 2), we divide the loop of *y*, instead of the loop of *β*. Usually, the number of cores on a PC is 2, 4 or 8, it is not common for *N*_*β*/4_ to be an integer, but it is always the case for *N*_*y*_ to be 256, 512, or 1024. Our parallel implementation on a multi-core PC is more efficient than that on a PC cluster in terms of time required for data exchange between threads. In our case, the data exchange is via on board RAM bus, while the PC cluster's data exchange via local network is significantly slower.

#### 2.2.5. Intel C++ compiling

The Intel C++ Compiler creates applications that can run at the fastest speeds on the Intel processors. It can take the full advantage of the Intel processors when compiling codes and generating object files. The Intel C++ compiler can be coupled with the Visual Studio. This provides an integrated development environment. In our implementation, we use it to optimize the code for Pentium D and Xeon processors.

## 3. NUMERICAL EXPERIMENTS

To test the gain of our scheme, we ran our accelerated code on Pentium D and Xeon PCs. Their configurations are listed in Table 1. Besides, different sizes of projection datasets and reconstructed images were tested to evaluate the efficiency under various conditions.

Here to test effeteness of each technique, the reconstruction times and speedups are tested by applying them gradually. The reconstruction experiments are done based on our HP6200 workstation and reconstructing a 512 × 512 image from a projection dataset (1160 × 672). The reconstruction results are shown by applying techniques step by step (Table 2). The overall speedup and individual speedups for each technique are also calculated to show the efficiency of them.

**Figure 10:**
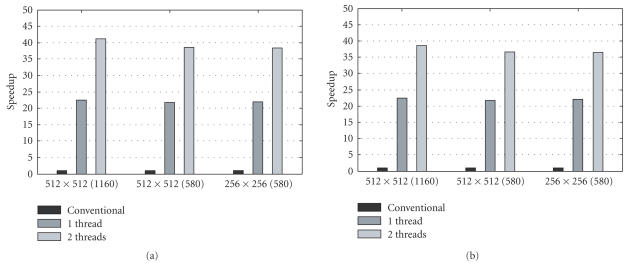
Experimental results on the speedup with our scheme: (a) results with the Pentium D PC and (b) with the Xeon PC.

The speedup results for different projection datasets and image matrix sizes are shown in Tables 3, 4, and Figure 10. The results on different computers are consistent. Significant speedups were achieved using our scheme. In the case of 1160 views and 512 × 512 image, the reconstruction time was decreased from 52 seconds to 1.35 seconds. For a one-core computer, the speedup was more than 20 times. For a two-core computer, the speedup was almost 40 times when 2 threads were used.

**Figure 11:**
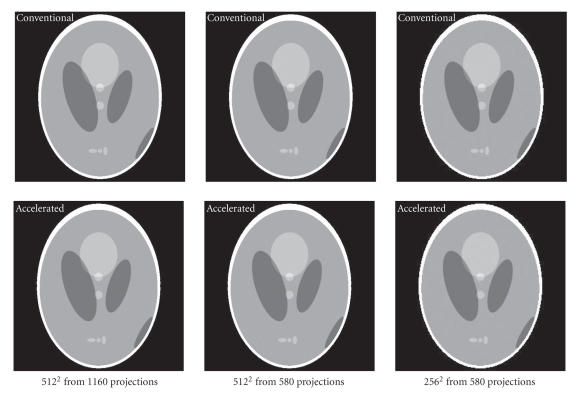
Reconstructed images of the Shepp-Logan using the conventional and accelerated codes (2 threads) in the display window [0.97, 1.05].

The Shepp-Logan head phantom was used in our numerical experiments. The images reconstructed using our scheme and the conventional method are shown in Figure 11. All the images were reconstructed using 32-bit floating-point data and were displayed in the same window [0.97, 1.05]. The images reconstructed using our accelerated and conventional schemes are essentially the same.

## 4. DISCUSSION AND CONCLUSIONS

As the CT reconstruction algorithm is highly parallelizable, the speedup can be improved with more cores almost linearly. For example, with two quad-core processors, the speedup that could be achieved is more than 100. As compared to other acceleration techniques, such as those based on specialized hardware and graphics cards, our general purpose PC-based scheme is much cheaper without compromising image quality. For example, a general purpose HP or Dell workstation with a top-line two quad-core processor and 8 GB RAM is less than $7000. All calculations are based on 32-bit floating point data, providing sufficient accuracy for medical imaging applications.

In terms of the absolute reconstruction time for a 512 × 512 image from 1160 projection views, it has been decreased from 52 to about 1.25 seconds. If the latest multi-core processor is used, the total time can be easily decreased by several folds. As the computers we have still use the previous generation processor, the potential improvement is at least 5 times if we are equipped with the latest quad-core processors [14], that is, the reconstruction time may be reduced to 0.3 second. Hence, it is quite promising for real-time CT applications, such as project on Bolus-chasing CT angiography.

In conclusion, our acceleration scheme has integrated several techniques including utilization of geometric symmetry, optimization of data structures, single-instruction multiple-data (SIMD) processing, multi-threaded computation, and an Intel C++ complier. As a result, it has speeded up the reconstruction process by 40 times, as compared to the conventional implementation on a general purpose PC with 2 cores. Further work is in progress to improve our results using the latest PCs and extend our scheme for cone-beam reconstruction.

## Figures and Tables

**Algorithm 1:**
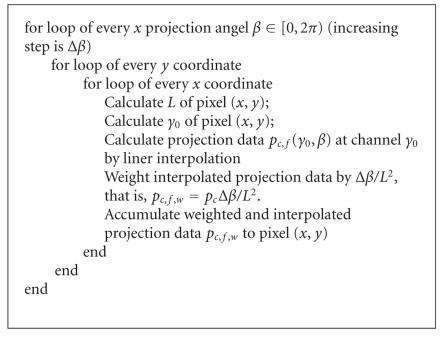
Pseudocode for the backprojection.

**Algorithm 2:**
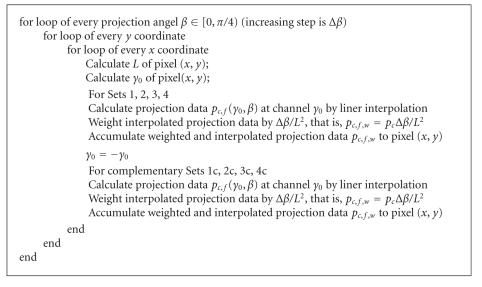
Pseudocode for the backprojection with the right-angle and complement symmetries.

**Table 1:** Configurations of the host computers.

Computer	Processor	RAM

HP 4300 workstation	One Pentium D 840 processor (3.2 GHz), two cores per processor, 90 nm chip	1.5 GB DDR2 RAM

HP 6200 workstation	Two Xeon 3.2 GHz processors, one core per processor, 90 nm chip	1.5 GB DDR2 RAM

**Table 2:** Reconstruction results by applying techniques gradually.

Acceleration techniques	None	Technique 1	Techniques 1, 2	Techniques 1–3	Techniques 1–4	Techniques 1–5

Reconstruction time (s)	51.5	7.25	6.12	4.91	2.62	1.25
Overall speedup	1	7.1	8.4	10.5	19.9	41.2

		Technique 1	Technique 2	Technique 3	Technique 4	Technique 5

Individual speedup	1	7.1	1.18	1.25	1.89	2.07

**Table 3:** Speedup comparison on HP 4300 workstation (one Pentium D 840 processor).

Projection data, *N*_projs_ × *N*_channels_	Image size	Optimized mode	Rebinning time (s)	Reconstruction time (s)	Total time (s)	Speedup

1160 × 672	512^2^	Conventional	0.029	51.5	51.5	1
		1 thread	0.029	2.26	2.289	22.5
		2 threads	0.029	1.224	1.25	41.2

580 × 672	512^2^	Conventional	0.016	25.78	25.8	1
		1 thread	0.016	1.162	1.18	21.8
		2 threads	0.016	0.655	0.671	38.5

580 × 672	256^2^	Conventional	0.016	7.65	7.67	1
		1 thread	0.016	0.342	0.33	21.9
		2 threads	0.015	0.185	0.20	38.4

**Table 4:** Speedup comparison on HP 6200 workstation (2 Xeon 3.2 GHz processors).

Projection data, *N*_projs_ × *N*_channels_	Image size	Optimized mode	Rebinning time (s)	Reconstruction time (s)	Total time (s)	Speed up

1160 × 672	512^2^	Conventional	0.032	52.0	52.0	1

		1 thread	0.032	2.273	2.31	22.5

		2 threads	0.032	1.315	1.35	38.5

580 × 672	512^2^	Conventional	0.0185	25.88	25.9	1

		1 thread	0.0185	1.167	1.19	21.7

		2 threads	0.0185	0.689	0.708	36.6

580 × 672	256^2^	Conventional	0.0185	7.67	7.69	1

		1 thread	0.0185	0.329	0.348	22.1

		2 threads	0.0185	0.1928	0.211	36.4
